# Bayesian network meta-analysis comparing hot balloon, laser balloon and cryoballoon ablation as initial therapies for atrial fibrillation

**DOI:** 10.3389/fcvm.2023.1184467

**Published:** 2023-07-25

**Authors:** Chenxia Wu, Luoxia Hu, Youjin Kong, Bowen Zhao, Wei Mao, Xinbin Zhou

**Affiliations:** ^1^ Department of Cardiology, The First Affiliated Hospital of Zhejiang Chinese Medical University (Zhejiang Provincial Hospital of Chinese Medicine), Hangzhou, China; ^2^ Department of Cardiology, Zhejiang Hospital, Hangzhou, China; ^3^ Key Laboratory of Integrative Chinese and Western Medicine for the Diagnosis and Treatment of Circulatory Diseases of Zhejiang Province, Hangzhou, China

**Keywords:** atrial fibrillation, cryoballoon ablation, hot balloon ablation, laser balloon ablation, network meta-analysis

## Abstract

**Background:**

Balloon-based catheter ablation (CA) technologies, including hot balloon ablation (HBA), laser balloon ablation (LBA) and cryoballoon ablation (CBA) have been introduced in recent years as alternatives to conventional radiofrequency ablation therapy for atrial fibrillation (AF). However, the results remain controversial concerning the optimal approach. Thus, we conducted a network meta-analysis (NMA) to comprehensively evaluate the efficacy and safety of HBA, LBA and CBA.

**Methods:**

Clinical trials comparing the efficacy and safety of HBA, LBA and CBA were identified through a systematic search up to October 2022. The primary outcomes of interest were the recurrence of AF and procedure-related complications.

**Results:**

Twenty clinical trials with a total of 1,995 patients were included in the meta-analysis. The NMA results demonstrated that HBA, LBA and CBA had comparable AF recurrence rates (HBA vs. CBA: odds ratio OR = 0.88, 95% credible interval CrI: 0.56–1.4; LBA vs. CBA: OR = 1.1, 95% CrI: 0.75–1.5; LBA vs. HBA: OR = 1.2, 95% CrI: 0.70–2.0) and procedure-related complications (HBA vs. CBA: OR = 0.93, 95% CrI: 0.46–2.3; LBA vs. CBA: OR = 1.1, 95% CrI: 0.63–2.1; LBA vs. HBA: OR = 1.2, 95% CrI: 0.44–2.8). The surface under the cumulative ranking curve (SUCRA) suggested that HBA may be the optimal approach concerning the primary outcomes (SUCRA = 74.4%; 61.1%, respectively). However, HBA (40.1%) had a significantly higher incidence of touch-up ablation (TUA) than LBA (8.5%, OR = 2.8, 95% CrI: 1.1–7.1) and CBA (11.9%, OR = 3.7, 95% CrI: 1.9–7.5). LBA required more procedure time than CBA [mean difference (MD = 32.0 min, 95% CrI: 19.0–45.0 min)] and HBA (MD = 26.0 min, 95% CrI: 5.6–45.0 min), but less fluoroscopy time than HBA (MD = −9.4 min, 95% CrI: −17.0–−2.4 min).

**Conclusions:**

HBA, LBA and CBA had comparable efficacy and safety as initial treatments for AF. HBA ranked highest in the primary outcomes, but at the cost of a higher incidence of TUA and longer fluoroscopy time.

**Systematic Review Registration:**

www.crd.york.ac.uk/prospero/display_record.php?ID=CRD42022381954, identifier: CRD42022381954.

## 1. Introduction

Atrial fibrillation (AF) is the most common sustained cardiac arrhythmia with increased risks of stroke and heart failure (1). Catheter ablation (CA) has been recommended as the first-line treatment option for patients with symptomatic AF in recent decades, with pulmonary vein isolation (PVI) being the standard treatment strategy (2).

Point-by-point radiofrequency ablation (RFA) has been commonly performed to achieve PVI; however, it still remains a complex and time-consuming procedure (3). To simplify PVI procedures, several balloon-based ablation technologies have been developed, including cryoballoon ablation (CBA), hot balloon ablation (HBA) and laser balloon ablation (LBA), which have rapidly emerged as alternatives to conventional CA for AF owing to their impressive procedural advantages and feasibility in several multicenter trials (3–5).

Several studies were conducted to compare the efficacy, safety, and procedural parameters between these three balloon-based CA treatments; however, the results were conflicting. Thus, we performed pairwise and Bayesian network meta-analyses to assess the efficacy, safety, and procedural characteristics between HBA, LBA and CBA and guide the optimal selection of these balloon-based strategies as initial treatments for AF.

## 2. Materials and methods

### 2.1. Search strategy and selection criteria

Databases including PubMed/MEDLINE, Embase, Web of Science, the Cochrane Library and ClinicalTrials.gov were systematically searched up to October 2022. The following terms and variants thereof were used: “hot balloon ablation”, “laser balloon ablation”, “cryoballoon ablation” and “atrial fibrillation”. In addition, the references of the selected articles and relevant reviews were manually searched for potentially relevant studies. To be included in our research, the studies were required to meet the following criteria: (1) published as a full-text article in English, (2) original data of comparisons between hot balloon, laser balloon and cryoballoon ablation as initial therapies for AF, and (3) the outcomes of interest had to be reported.

### 2.2. Data collection and quality assessment

Two investigators (LXH and YJK) independently extracted data from studies and assessed the quality. Discrepancies were resolved by consensus with a third investigator (XBZ). The following data were extracted: study and participant characteristics, ablation strategy, intervention-related data, duration of follow-up, and outcomes of interest. The quality of the included randomized controlled trials (RCTs) was assessed with the Cochrane Collaboration tool (6), and the nonrandomized studies were evaluated using the ROBINS-I tool (7).

### 2.3. Primary and secondary outcomes

The primary outcomes of interest were the recurrence of AF and procedure-related complications. AF recurrence was defined as atrial tachyarrhythmias (AT), including AF, atrial flutter or atrial tachycardia documented on the ECG or Holter continuing longer than 30 s after the CA procedure during follow-up. Procedure-related complications were defined as major complications, including death, cardiac tamponade, stroke, symptomatic PV stenosis and persistent phrenic nerve palsy (PNP), and minor complications, including transient ischemic attack (TIA), transient PNP and vascular complications. Secondary outcomes included the touch-up ablation (TUA) rate, total procedure time and fluoroscopy time. The TUA was defined as additional touch-up radiofrequency catheter ablation for residual/dormant PV conduction to achieve PVI, if PVI could not be achieved after HBA, LBA or CBA.

### 2.4. Statistical analysis

Continuous variables were described as median and standard deviation (SD), and categorical variables were described as *n* (%). STATA version 14.0 (STATA Corporation, College Station, TX, USA) was applied to perform a pairwise meta-analysis. The odds ratio (OR), weighted mean difference (WMD) and the 95% confidence interval (CI) were calculated to demonstrate the overall result.

For indirect and mixed comparisons, a Bayesian network meta-analysis was performed using *R* version 3.6.2 with GeMTC packages computing OR or mean difference (MD) and their 95% credible interval (CrI). Markov chain Monte Carlo (MCMC) methods were applied to sample posterior probabilities with Gibbs sampling from 100,000 iterations. The relative ranking was assessed with the surface under the cumulative ranking curve (SUCRA) probabilities. The SUCRA is a summary of the rank distribution which can be interpreted as the estimated proportion of treatments worse than the treatment of interest, and a SUCRA with a value of 100% indicated that the treatment would be the best.

Pairwise heterogeneity across studies was assessed with the chi-square test, and *I*^2^ > 50% indicated significant heterogeneity. When heterogeneity was present, the possible causes were investigated. Network inconsistency was evaluated using the previously described node-splitting method comparing the results derived from consistent and inconsistent models (8). Publication bias was analyzed graphically by funnel plots and statistically by Egger’s and Begg’s tests. This study is reported in accordance with the Preferred Reporting Items for Systematic Reviews and Meta-Analyses (PRISMA) guidelines, and the protocol was registered at PROSPERO (doi: 10.15124/CRD42022381954).

## 3. Results

### 3.1. Eligible studies and characteristics

**Figure 1:**
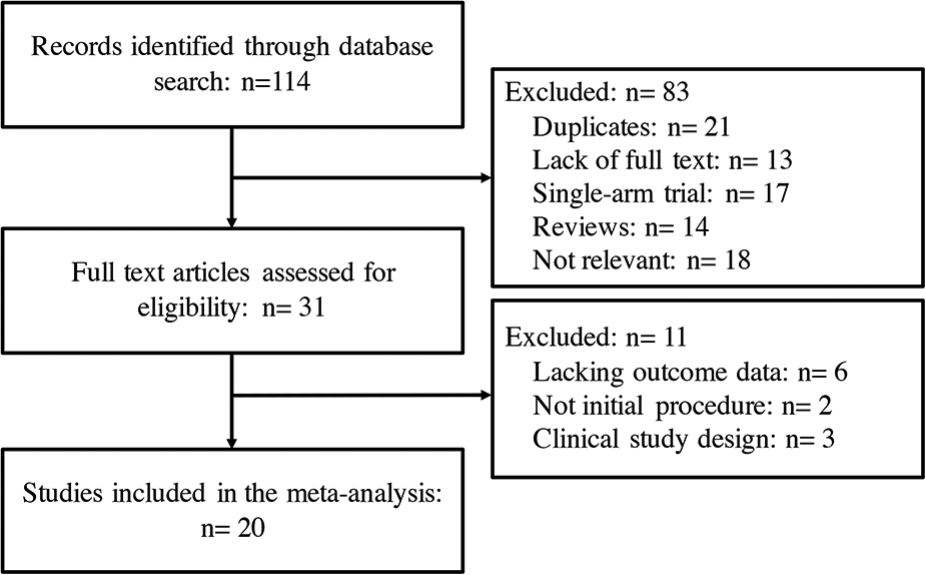
Flow chart of the systematic literature research.

A total of 114 potentially relevant studies were identified in the initial search, of which 31 studies were further assessed. Finally, 20 clinical trials (9–28) with a total of 1,995 patients were included in the meta-analysis (Figure 1). No additional studies were identified. The baseline characteristics of the included studies are presented in Table 1. Briefly, across the trials, three studies were RCTs (11, 12, 20), while the remaining studies were nonrandomized trials, including eleven prospective studies and six retrospective studies. Seven studies (9, 13, 16, 17, 23, 25, 26) compared HBA with CBA, eleven studies (10–12, 15, 18–20, 22, 24, 27, 28) compared LBA with CBA, and two studies (14, 21) compared HBA, LBA and CBA simultaneously. Eight studies included only paroxysmal AF (PAF) patients, and the remaining 12 studies included both PAF and persistent AF (PerAF) patients. In total, 436 patients were in the HBA group, 603 patients were in the LBA group, and 956 patients were in the CBA group. The mean follow-up length across studies was 12 months. All included studies were of good quality according to the Cochrane Collaboration tool (6) and ROBINS-I tool (7). No significant publication bias was found by funnel plot or Egger’s and Begg’s tests based on the primary outcomes (Egger’s: *p* = 0.312; Begg’s: *p* = 0.976).

**Table 1:** Baseline characteristics of the included studies.

Study	Year	Study type	*N*	PAF (%)	Mean age (years)	Male (%)	Mean LVEF(%)	Mean LAd (mm)	DM (%)	Hypertension (%)	HF (%)	Treatment	Follow-up
Suruga (23)	2022	Retrospective	60	100	63.5	85	65	39.5	21.67	48.33	8.33	HBA vs. CBA	12 m
Hojo (13)	2020	Prospective	92	100	65.15	81.52	64.5	NR	NR	NR	NR	HBA vs. CBA	6 m
Wakamatsu (26)	2020	Retrospective	158	0	64	77.2	62	41	16.5	53.2	13.9	HBA vs. CBA	18 m
Akita (9)	2019	Retrospective	80	93.8	64.6	78.8	62	38.5	NR	NR	NR	HBA vs. CBA	12 m
Nakamura (17)	2019	Prospective	123	95.1	65	68.3	63	38	17.1	46.3	3.3	HBA vs. CBA	46 days
Wakamatsu (25)	2019	Retrospective	92	63	62.5	76.1	66	39.5	19.6	48.9	6.5	HBA vs. CBA	12 m
Nagashima (16)	2018	Retrospective	74	62	62	74.3	66	40	24.3	59.5	9.5	HBA vs. CBA	12 m
Schiavone (19)	2022	Prospective	110	57.3	63.2	68.2	61	NR	8.2	69.1	4.6	LBA vs. CBA	12 m
Chun (12)	2021	RCT	200	50	65.8	56	61.5	39.5	10.5	66.5	1.5	LBA vs. CBA	12 m
Yano (28)	2021	Prospective	111	100	73	57.7	70	42.5	14.4	58.6	7.2	LBA vs. CBA	350 days
Perrotta (18)	2017	Prospective	40	80	66.5	73	62	40	5	88	NR	LBA vs. CBA	12 m
Stockigt (22)	2016	Retrospective	70	0	65.2	77	55.2	NR	19	79	NR	LBA vs. CBA	12 m
Tsyganov (24)	2015	Prospective	100	100	62.5	63	NR	43	16	64	NR	LBA vs. CBA	12 m
Casella (11)	2014	RCT	55	100	57.6	72.7	61.8	41.8	NR	40	NR	LBA vs. CBA	12 m
Wissner (27)	2014	Prospective	64	83.3	62.4	67	64	42.4	5	69	NR	LBA vs. CBA	6 m
Kumar (15)	2014	Prospective	60	88.3	65	73	57.7	NR	NR	NR	NR	LBA vs. CBA	302 days
Schmidt (20)	2013	RCT	66	100	65.5	NR	60	40	6.1	75.8	NR	LBA vs. CBA	21 m
Bordignon (10)	2013	Prospective	140	100	63	66	63	39.9	12	62	NR	LBA vs. CBA	12 m
Seki (21)	2022	Prospective	150	82.7	66.6	70.7	58.3	38.7	8.7	43.3	NR	HBA vs. LBA vs. CBA	21 m
Kobori (14)	2021	Prospective	150	100	65.4	72	62.6	36.3	NR	51.3	5.3	HBA vs. LBA vs. CBA	12 m

PAF, paroxysmal atrial fibrillation; LVEF, left ventricular ejection fraction; Lad, left atrial diameter; DM, diabetes mellitus; HF, heart failure; NR, not reported; HBA, hot balloon ablation; LBA, laser balloon ablation; CBA, cryoballoon ablation; RCT, randomized controlled trial.

### 3.2. Primary endpoints

#### 3.2.1. AF recurrence

Of the included trials, 17 studies (9–16, 18, 19, 21–26, 28) reported on the AF recurrence rate after CA. A pairwise meta-analysis demonstrated that the AF recurrence rate did not significantly differ between the HBA (13.5%), LBA (30.0%) and CBA groups (21.5%) (HBA vs. CBA: OR = 0.83, 95% CI: 0.55–1.25, *p* = 0.367; LBA vs. CBA: OR = 1.07, 95% CI: 0.77–1.49, *p* = 0.683; HBA vs. LBA: OR = 1.08, 95% CI: 0.50–2.31, *p* = 0.847). No significant heterogeneity was detected for the comparisons (*I*^2^ = 0%, 23.3%, 0%, respectively) (Supplementary Figure S1).

**Figure 2:**
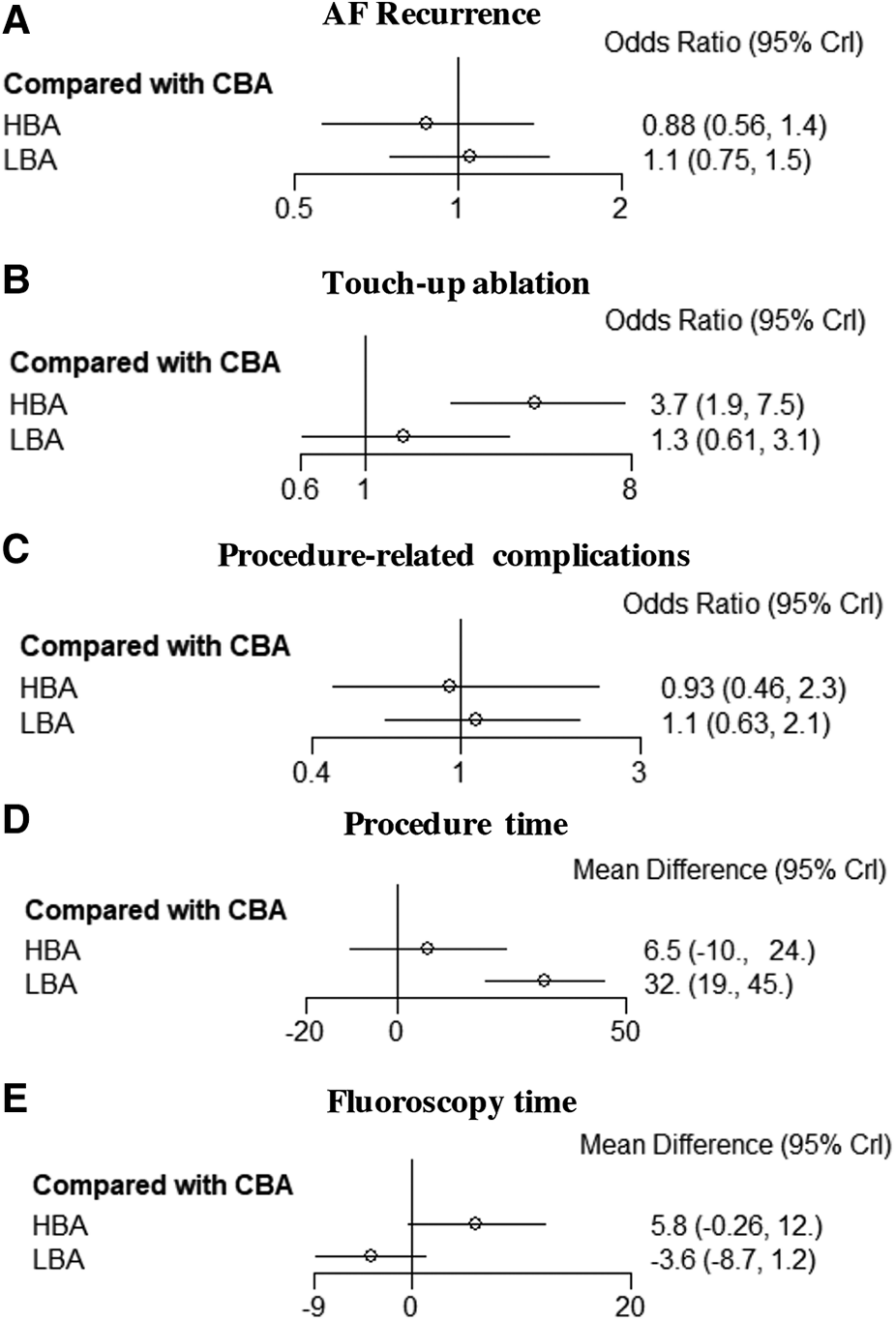
Forest plots of network meta-analysis for the primary and secondary outcomes. (**A**) AF recurrence, (**B**) Touch-up ablation, (**C**) Procedure-related complications, (**D**) Procedure time, (**E**) Fluoroscopy time. AF, atrial fibrillation; HBA, hot balloon ablation; LBA, laser balloon ablation; CBA, cryoballoon ablation.

**Figure 3:**
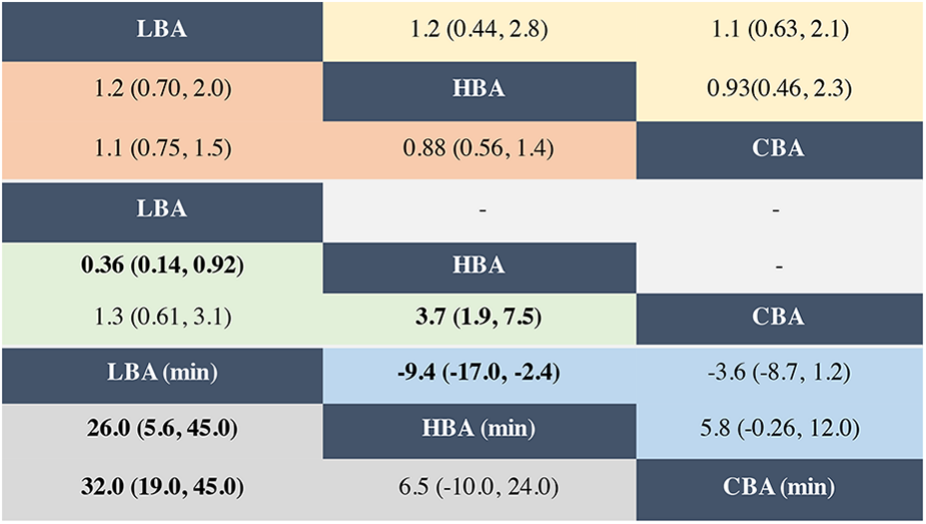
Ors and MDs with their 95% Crls of network meta-analysis for the primary and secondary outcomes. Results of network meta-analysis for AF recurrence (in orange), procedure-related complications (in yellow), TUA (in green), procedure time (in grey) and fluoroscopy time (in blue) were listed in the triangles, and the estimation was calculated as the column-defining treatment compared with the row-defining treatment. Bold font indicates the difference was statistically significant. LBA, laser balloon ablation; HBA, hot balloon ablation; CBA, cryoballoon ablation; OR, odds ratio; MD, mean difference; TUA, touch-up ablation.

**Figure 4:**
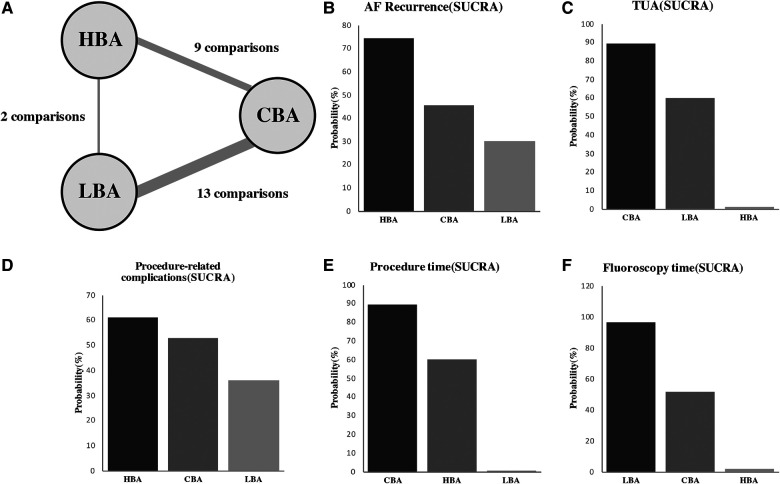
Ranking probabilities according to SUCRA. (**A**) Evidence structure of direct comparisons included for network meta-analysis. The thickness of the line corresponds to the number of comparisons; Ranking probabilities between HBA, CBA and LBA for the outcomes including (**B**) AF recurrence, (**C**) TUA, (**D**) procedure-related complications, (**E**) procedure time, (**F**) fluoroscopy time. HBA, hot balloon ablation; LBA, laser balloon ablation; CBA, cryoballoon ablation; AF, atrial fibrillation; SUCRA, surface under the cumulative ranking curve.

The NMA results also showed that compared with CBA, HBA (OR = 0.88, 95% CrI: 0.56–1.4) and LBA (OR = 1.1, 95% CrI: 0.75–1.5) had comparable AF recurrence rates (Figure 2). There were no significant differences in the AF recurrence rate between the three balloon-base strategies (Figure 3). The network of the primary outcomes is shown in Figure 4. The SUCRA suggested that HBA may be the optimal approach (SUCRA = 74.4%), followed by CBA (SUCRA = 45.5%) and LBA (SUCRA = 30.1%) (Figure 4).

#### 3.2.2. Procedure-related complications

Eighteen trials (9–22, 24, 26–28) reported on the outcome of procedure-related complications. Pairwise meta-analysis showed comparable complication rates between the HBA (7.2%), LBA (7.8%) and CBA groups (7.8%) (HBA vs. CBA: OR = 0.73, 95% CI: 0.40–1.34, *p* = 0.300; LBA vs. CBA: OR = 1.02, 95% CI: 0.63–1.65, *p* = 0.931; HBA vs. LBA: OR = 0.85, 95% CI: 0.27–2.63, *p* = 0.774). No significant heterogeneity was detected for the comparisons (*I*^2^ = 4.2%, 5.3%, 0%, respectively) (Supplementary Figure S2).

The NMA results showed that compared with CBA (SUCRA = 52.9%), HBA (OR = 0.93, 95% CrI: 0.46–2.3, SUCRA = 61.1%) and LBA (OR = 1.1, 95% CrI: 0.63–2.1, SUCRA = 36.0%) had comparable AF recurrence rates (Figure 2). There were no significant differences concerning procedure-related complications between the three ablation strategies (Figure 3).

Deviance information criterion (DIC) value analysis was applied to compare the model fit of the NMA models with and without the assumption of evidence inconsistency. The results demonstrated good consistency with similar DIC values between the two models concerning the primary endpoints (DIC: 55.04, *I*^2^ = 0% vs. DIC: 55.21, *I*^2^ = 0%).

### 3.3. Secondary endpoints

#### 3.3.1. TUA

Seventeen trials (9–17, 19, 21–27) reported on the TUA rate. A pairwise meta-analysis demonstrated that HBA (40.1%) had a significantly higher TUA rate than CBA (11.9%, OR = 3.26, 95% CI: 2.22–4.77, *p* = 0.00) and LBA (8.5%, OR = 4.82, 95% CI: 2.38–9.77, *p *= 0.00). No significant heterogeneity was detected (*I*^2^ = 24.6%, and 33.2%, respectively). LBA and CBA had comparable TUA rates (OR = 1.73, 95% CI: 0.81–3.72, *p* = 0.159) (Supplementary Figure S3).

The NMA results also showed that, compared with CBA, HBA had a significantly higher TUA rate (OR = 3.7, 95% CrI: 1.9–7.5), while LBA had comparable results (OR = 1.3, 95% CrI: 0.61–3.1). LBA had significantly lower TUA rates than HBA (OR = 0.36, 95% CrI: 0.14–0.92) (Figures 2, 3). The SUCRA suggested that CBA (SUCRA = 89.3%) may need the least TUA (SUCRA = 74.4%), followed by LBA (SUCRA = 59.7%), while HBA needed the most TUA during the procedure (SUCRA = 1.0%) (Figure 4).

#### 3.3.2. Procedure time

Seventeen studies (9, 10, 12–15, 17–24, 26–28) provided data regarding procedure time. A pairwise meta-analysis demonstrated that HBA and CBA had comparable procedure times (WMD = 6.68 min, 95% CI: −4.30–17.65 min, *p* = 0.223); LBA needed significantly more procedure time than CBA (WMD = 31.56 min, 95% CI: 17.85–45.27 min, *p* = 0.00) and HBA (WMD = 31.94 min, 95% CI: 18.26–45.62 min, *p* = 0.00). However, moderate to significant heterogeneities were detected (Supplementary Figure S4).

The NMA results also showed similar results: LBA (SUCRA = 0.4%) required more procedure time than CBA (MD = 32.0 min, 95% CrI: 19.0–45.0 min, SUCRA = 89.3%) and HBA (MD = 26.0 min, 95% CrI: 5.6–45.0 min, SUCRA = 60.3%) (Figures 2–4).

#### 3.3.3. Fluoroscopy time

Thirteen studies (9, 10, 12–15, 18, 19, 21–24, 26) provided data regarding fluoroscopy time. A pairwise meta-analysis demonstrated that HBA had comparable fluoroscopy time compared with CBA (WMD = 4.87 min, 95% CI: −1.35–11.09 min, *p* = 0.125) and LBA (WMD = 13.51 min, 95% CI: −6.48–33.49 min, *p* = 0.185), while LBA had significantly less fluoroscopy time than CBA (WMD = −3.44 min, 95% CI: −6.52–−0.36 min, *p* = 0.029). However, significant heterogeneities were detected for these three comparisons (Supplementary Figure S5).

However, the NMA results showed that compared with CBA, HBA (MD = 5.8 min, 95% CrI: −0.26–12 min) and LBA (MD = −3.6 min, 95% CrI: −8.7–1.2 min) had comparable fluoroscopy times; LBA required significantly less fluoroscopy time than HBA (MD = −9.4 min, 95% CrI: −17.0–−2.4 min) (Figures 2, 3). The SUCRA suggested that, LBA may be the optimal approach (SUCRA = 96.6%) concerning fluoroscopy time, followed by CBA (SUCRA = 51.6%) and HBA (SUCRA = 1.8%) (Figure 4).

In addition, we further investigated the reasons for the different results between pairwise meta-analysis and NMA. The results showed that when the standard mean difference (SMD) was calculated in the pairwise meta-analysis, LBA and CBA had comparable fluoroscopy times (SMD = −0.32 min, 95% CI: −0.69–0.05 min, *p* = 0.09), while HBA had significantly more fluoroscopy time than LBA (SMD = 0.83 min, 95% CI: 0.03–1.64 min, *p* = 0.042).

## 4. Study limitations

The present study used both pairwise and network meta-analytic methods based on 20 trials with 1,995 patients; however, there are several limitations. First, only three trials were RCTs, and most evidence was from nonrandomized trials. Although the quality of the included studies was adequate, blinding of patients and the operators was not possible. Second, a mixed population of AF (PAF and PerAF) was included, and subgroup analysis was not available due to the lack of certain data. Third, the follow-up period of some included studies was relatively short, which could have influenced the clinical outcomes. Finally, SUCRA was calculated to rank each treatment option in the NMA, whereas, SUCRA does not measure the magnitude of difference between treatments. Thus, the results should be interpreted with a combination of the estimated OR/WMD and 95% Cr/CrI.

## 5. Discussion

In recent years, balloon-based CA strategies, including HBA, CBA and LBA, have rapidly emerged as alternative therapeutic approaches to conventional point-by-point RFA therapy for the initial treatment of AF (3–5). However, the best treatment option remains unclear. To the best of our knowledge, the present study is the first network meta-analysis to comprehensively compare the clinical efficacy, safety, and procedural parameters between these three balloon-based CA modalities and to rank these treatment strategies for guiding the optimal selection. The principal findings were as follows: HBA, LBA and CBA had comparable rates of AF recurrence and procedure-related complications; SUCRA suggested that HBA may be the optimal approach concerning the primary outcomes; HBA had a significantly higher incidence of TUA than LBA and CBA. LBA required more procedure time than CBA and HBA, but less fluoroscopy time than HBA.

Balloon-based CA technologies have the advantage of reducing the complexity of point-by-point RFA procedures by placing the balloon catheter at the antrum/ostium of the PV (3). Based on evidence from both pairwise and network meta-analyses, we found no significant difference in terms of AF recurrence between the three techniques. These results are consistent with the previously published pairwise meta-analysis, which demonstrated comparable AF recurrence rates during the mid-term follow-up between HBA and CBA (29).

CBA is currently the most common alternative therapy to RFA (14). Plenty of evidence from RCTs, including the large-scale Fire and ICE trial, showed the high effectiveness of CBA as first-line therapy for AF patients, especially for symptomatic PAF (3, 19). LBA and HBA were mainly applied in JAPAN, and LBA has the advantage of providing endoscopic visualization of the endocardial surface with a compliant balloon (30). A prospective, multicenter, randomized trial showed non-inferior safety and efficacy profiles of LBA to RFA (4). However, as the most recent proposed technology, little evidence has been reported regarding the direct comparisons between HBA and RFA. The only RCT that was proposed in 2016 showed the superiority of HBA compared with antiarrhythmic drug therapy for the treatment of patients with PAF (5). However, when compared with CBA and LBA, HBA demonstrated the best rank probability in terms of AF recurrence, although TUA was most frequently needed. This may suggest that the SUCRA recurrence rate preference of HBA is the result of a hybrid procedure.

Similar to the efficacy endpoint, comparable risk estimates for procedure-related complications were found between the three ablation techniques, ranging from 7.2% to 7.8%. Common major complications included PNP, cardiac tamponade and pulmonary vein stenosis (13, 14). The prevalence of major complications after CA for AF varies from 0.8% to 16.3% (31). The study by Chun et al. evaluated the complications in CA of AF in 3,000 patients and showed that cardiac tamponade occurred in 0.1% of the patients treated with balloon catheters, which was significantly lower than RFA (1.5%) (32). This study indicated a low chance of cardiac tamponade for balloon-based ablation therapy, and the overall risk for balloon-based technique-specific complications, such as PNP, was reported to be approximately 1.5% (32). PNP represents a relatively common complication for balloon-based therapy; however, most PNP cases are usually transient, and persistent PNP occurs in <1% of patients, who usually recover function after 3 months (12, 33).

It should be noted that the estimated risks of procedure-related complications were higher than those reported by the abovementioned studies (32). A possible explanation might be that all the reported complications from the included studies were evaluated; however, the definition of procedure-related complications was relatively heterogeneous among the included studies. Complications, such as minor vascular complications, transient ischemic attacks, and gastroparesis, were also defined as procedure-related complications in several included studies.

Although the clinical outcomes in terms of efficacy and safety are comparable between the HBA, CBA and LBA techniques, HBA required a significantly higher incidence of additional TUA. This unequal phenomenon may be attributed to the inherent characteristics of HBA. It was reported that, HB had better adjustability and compliance than CB during the procedure, which could help to occlude the more distal and deeper portions of the PVs (16, 34). However, this elastic feature may lead to inadequate occlusion of the PV antrum which may not adhere well to all the tissues of the PV walls (25). Compared to the HB, the nonconforming CB stiffens the balloon during energy deliveries, creating a larger ablation area of the PV antrum (9). Several studies have demonstrated that CBA produced a wider and larger lesion width than HBA (9, 16). Therefore, the relatively smaller LA lesion areas created by HBA compared with CBA may lead to a higher incidence of TUA.

Another possible explanation could be that the surface temperature of the hot balloon may not be higher enough to cause deep tissue damage. A previous study by Hojo et al. showed that a balloon temperature of 73°C yielded a lower TUA rate than that at 70°C. The thickest anterior aspect of the left superior pulmonary vein (LSPV) was reported to be the dominant TUA area (13, 23). However, a higher balloon temperature may increase the risk of PV stenosis; thus, additional TUA is still recommended for HBA (13). Even though HBA had the highest rates of additional TUA compared with CBA and LBA, the AF recurrence rates between these techniques were comparable, suggesting benefit from the deeper lesions in the PVs. Technical developments to improve the contact between the HB and PV tissue are warranted to reduce TUA rates.

In addition, HBA may need the most fluoroscopy time according to the SUCRA, while LBA may have the least fluoroscopy time. It was reasonable to expect that LBA needed the lowest fluoroscopy time given the use of an endoscopic guide, while CBA and HBA require serial angiograms to achieve optimal PV occlusion (14). It should be noted that, the results were inconsistent between the pairwise and network meta-analyses in terms of fluoroscopy time analysis. NWM demonstrated that LBA and CBA had comparable fluoroscopy times but had less fluoroscopy time than HBA (Figure 2). However, a pairwise meta-analysis showed that, LBA had less fluoroscopy time than CBA and had comparable results compared with HBA (Supplementary Figure S5). Potential causes were investigated, and we found that when the effect quantities for pairwise meta-analysis were changed from WMD to SMD, the same results were obtained between pairwise and network meta-analyses. The relatively small sample size and the high heterogeneities between studies may be possible explanations.

Another secondary endpoint, procedural time, should also be considered, as the three balloon-based strategies had similar efficacy and safety. Both pairwise and network meta-analyses demonstrated that HBA and CBA had comparable procedural times, but LBA needed the most procedural time according to SUCRA. LBA had the advantage of providing direct PV visualization to achieve a more precise titration of ablation lesions, whereas this would consume more procedure time than the “single shot” techniques, such as HBA and CBA (35). In addition, in contrast to CBA, LBA lacks a certain mapping catheter integrated into the system for real-time PV potential recording, leading to the increased procedural time when validating PVI using an additional mapping catheter (12). New generations of LB have been introduced recently, which showed promising improvements in PV occlusion characteristics and significantly decreased procedural and total laser times (36). Thus, as newly established CA techniques for AF, balloon-based ablation strategies have provided electrophysiologists with impressive alternative options. Larger prospective multicenter randomized studies with long-term follow-up are needed to compare these three balloon-based ablation techniques.

## 6. Conclusions

The present NMA showed that AF recurrence and procedure-related complications after CA for AF are comparable between HBA, LBA and CBA, while HBA showed the best rank. However, HBA required significantly more TUA during the procedure than CBA and LBA. The HBA strategy ranked highest with regard to procedure time (least), while the LBA strategy ranked highest with regard to fluoroscopy time (least). This study provides decision-makers with comprehensive and comparative evidence about the efficacy and safety of different balloon-based CA strategies. Further large-scale studies are still warranted to provide an up-to-date recommendation for the superior option.

## Data Availability

The raw data supporting the conclusions of this article will be made available by the authors, without undue reservation.

## Supplementary material

Supplementary Figure 1 Forest plot for the outcome of AF recurrence from pairwise meta-analysis. AF, atrial fibrillation; HBA, hot balloon ablation; LBA, laser balloon ablation; CBA, cryoballoon ablation; OR odds ratio.

Supplementary Figure 2 Forest plot for the outcome of procedure-related complication from pairwise meta-analysis. HBA, hot balloon ablation; LBA, laser balloon ablation; CBA, cryoballoon ablation; OR, odds ratio.

Supplementary Figure 3 Forest plot for the outcome of TUA from pairwise meta-analysis. HBA, hot balloon ablation; LBA, laser balloon ablation; CBA, cryoballoon ablation; OR, odds ratio; TUA, touch-up ablation.

Supplementary Figure 4 Forest plot for the outcome of procedure time from pairwise meta-analysis. HBA, hot balloon ablation; LBA, laser balloon ablation; CBA, cryoballoon ablation; WMD, weighted mean difference.

Supplementary Figure 5 Forest plot for the outcome of fluoroscopy time from pairwise meta-analysis. HBA, hot balloon ablation; LBA, laser balloon ablation; CBA, cryoballoon ablation; WMD, weighted mean difference.

## References

[1] Schnabel RB Yin X Gona P Larson MG Beiser AS McManus DD 50 year trends in atrial fibrillation prevalence, incidence, risk factors, and mortality in the Framingham heart study: a cohort study. Lancet. (2015) 386:154–62. 10.1016/s0140-6736(14)61774-8 25960110 PMC4553037

[2] Kirchhof P Benussi S Kotecha D Ahlsson A Atar D Casadei B 2016 ESC guidelines for the management of atrial fibrillation developed in collaboration with EACTS. Europace. (2016) 18:1609–78. 10.1093/europace/euw295 27567465

[3] Kuck KH Brugada J Furnkranz A Metzner A Ouyang F Chun KR Cryoballoon or radiofrequency ablation for paroxysmal atrial fibrillation. N Engl J Med. (2016) 374:2235–45. 10.1056/NEJMoa1602014 27042964

[4] Dukkipati SR Cuoco F Kutinsky I Aryana A Bahnson TD Lakkireddy D Pulmonary vein isolation using the visually guided Laser balloon: a prospective, multicenter, and randomized comparison to standard radiofrequency ablation. J Am Coll Cardiol. (2015) 66:1350–60. 10.1016/j.jacc.2015.07.036 26383722

[5] Sohara H Ohe T Okumura K Naito S Hirao K Shoda M Hotballoon ablation of the pulmonary veins for paroxysmal AF: a multicenter randomized trial in Japan. J Am Coll Cardiol. (2016) 68:2747–57. 10.1016/j.jacc.2016.10.037 28007137

[6] Higgins JP Altman DG Gøtzsche PC Jüni P Moher D Oxman AD The cochrane collaboration’s tool for assessing risk of bias in randomised trials. Br Med J. (2011) 343:d5928. 10.1136/bmj.d5928 22008217 PMC3196245

[7] Sterne JA Hernán MA Reeves BC Savović J Berkman ND Viswanathan M ROBINS-I: a tool for assessing risk of bias in non-randomised studies of interventions. Br Med J. (2016) 355:i4919. 10.1136/bmj.i4919 27733354 PMC5062054

[8] Dias S Welton NJ Caldwell DM Ades AE. Checking consistency in mixed treatment comparison meta-analysis. Stat Med. (2010) 29:932–44. 10.1002/sim.3767 20213715

[9] Akita T Kiuchi K Fukuzawa K Shimane A Matsuyama S Takami M Lesion distribution after cryoballoon ablation and hotballoon ablation: late-gadolinium enhancement magnetic resonance imaging analysis. J Cardiovasc Electrophysiol. (2019) 30:1830–40. 10.1111/jce.14073 31310389

[10] Bordignon S Chun KR Gunawardene M Fuernkranz A Urban V Schulte-Hahn B Comparison of balloon catheter ablation technologies for pulmonary vein isolation: the laser versus cryo study. J Cardiovasc Electrophysiol. (2013) 24:987–94. 10.1111/jce.12192 23800359

[11] Casella M Dello Russo A Russo E Al-Mohani G Santangeli P Riva S Biomarkers of myocardial injury with different energy sources for atrial fibrillation catheter ablation. Cardiol J. (2014) 21:516–23. 10.5603/CJ.a2013.0153 24293166

[12] Chun JKR Bordignon S Last J Mayer L Tohoku S Zanchi S Cryoballoon versus laserballoon: insights from the first prospective randomized balloon trial in catheter ablation of atrial fibrillation. Circ Arrhythm Electrophysiol. (2021) 14:e009294. 10.1161/circep.120.009294 33417476

[13] Hojo R Fukamizu S Tokioka S Inagaki D Miyazawa S Kawamura I Comparison of touch-up ablation rate and pulmonary vein isolation durability between hot balloon and cryoballoon. J Cardiovasc Electrophysiol. (2020) 31:1298–306. 10.1111/jce.14485 32270566

[14] Kobori A Sasaki Y Pak M Okada T Toyota T Kim K Early experiences with three types of balloon-based ablation catheters in patients with paroxysmal atrial fibrillation. Heart Rhythm O2. (2021) 2:223–30. 10.1016/j.hroo.2021.03.009 34337572 PMC8322794

[15] Kumar N Blaauw Y Timmermans C Pison L Vernooy K Crijns H. Adenosine testing after second-generation balloon devices (cryothermal and laser) mediated pulmonary vein ablation for atrial fibrillation. J Interv Card Electrophysiol. (2014) 41:91–7. 10.1007/s10840-014-9921-z 25012971

[16] Nagashima K Okumura Y Watanabe I Nakahara S Hori Y Iso K Hot balloon versus cryoballoon ablation for atrial fibrillation: lesion characteristics and middle-term outcomes. Circ Arrhythm Electrophysiol. (2018) 11:e005861. 10.1161/circep.117.005861 29700055

[17] Nakamura K Sasaki T Take Y Okazaki Y Inoue M Motoda H Postablation cerebral embolisms in balloon-based atrial fibrillation ablation with periprocedural direct oral anticoagulants: a comparison between cryoballoon and hotballoon ablation. J Cardiovasc Electrophysiol. (2019) 30:39–46. 10.1111/jce.13762 30288849

[18] Perrotta L Konstantinou A Bordignon S Fuernkranz A Dugo D Chun KJ What is the acute antral lesion size after pulmonary vein isolation using different balloon ablation technologies? Circ J. (2017) 81:172–9. 10.1253/circj.CJ-16-0345 27980294

[19] Schiavone M Gasperetti A Montemerlo E Pozzi M Sabato F Piazzi E Long-term comparisons of atrial fibrillation ablation outcomes with a cryoballoon or laser-balloon: a propensity-matched analysis based on continuous rhythm monitoring. Hellenic J Cardiol. (2022) 65:1–7. 10.1016/j.hjc.2022.03.006 35331905

[20] Schmidt B Gunawardene M Krieg D Bordignon S Fürnkranz A Kulikoglu M A prospective randomized single-center study on the risk of asymptomatic cerebral lesions comparing irrigated radiofrequency current ablation with the cryoballoon and the laser balloon. J Cardiovasc Electrophysiol. (2013) 24:869–74. 10.1111/jce.12151 23601001

[21] Seki R Nagase T Asano S Fukunaga H Inoue K Sekiguchi Y Radiofrequency current versus balloon-based ablation for atrial fibrillation. Am J Cardiol. (2022) 178:52–9. 10.1016/j.amjcard.2022.05.029 35817597

[22] Stöckigt F Kohlmann AT Linhart M Nickenig G Andrié RP Beiert T Laserballoon and cryoballoon pulmonary vein isolation in persistent and longstanding persistent atrial fibrillation. Pacing Clin Electrophysiol. (2016) 39:1099–107. 10.1111/pace.12929 27484618

[23] Suruga K Suenari K Nakano T Takemoto H Hashimoto Y Tomomoi S Comparison between cryoballoon and hot balloon ablation in patients with paroxysmal atrial fibrillation. J Interv Card Electrophysiol. (2022) 64:281–90. 10.1007/s10840-021-00978-0 33728551

[24] Tsyganov A Petru J Skoda J Sediva L Hala P Weichet J Anatomical predictors for successful pulmonary vein isolation using balloon-based technologies in atrial fibrillation. J Interv Card Electrophysiol. (2015) 44:265–71. 10.1007/s10840-015-0068-3 26475792

[25] Wakamatsu Y Nagashima K Nakahara S Iso K Watanabe R Arai M Electrophysiologic and anatomic factors predictive of a need for touch-up radiofrequency application for complete pulmonary vein isolation: comparison between hot balloon- and cryoballoon-based ablation. J Cardiovasc Electrophysiol. (2019) 30:1261–9. 10.1111/jce.13989 31111558

[26] Wakamatsu Y Nakahara S Nagashima K Fukuda R Nishiyama N Watanabe R Hot balloon versus cryoballoon ablation for persistent atrial fibrillation: lesion area, efficacy, and safety. J Cardiovasc Electrophysiol. (2020) 31:2310–8. 10.1111/jce.14646 32613693

[27] Wissner E Metzner A Neuzil P Petru J Skoda J Sediva L Asymptomatic brain lesions following laserballoon-based pulmonary vein isolation. Europace. (2014) 16:214–9. 10.1093/europace/eut250 23933850

[28] Yano M Egami Y Ukita K Kawamura A Nakamura H Matsuhiro Y Impact of myocardial injury and inflammation due to ablation on the short-term and mid-term outcomes: cryoballoon versus laser balloon ablation. Int J Cardiol. (2021) 338:102–8. 10.1016/j.ijcard.2021.06.016 34126131

[29] Peng X Liu X Tian H Chen Y Li X. Effects of hot balloon vs. Cryoballoon ablation for atrial fibrillation: a systematic review, meta-analysis, and meta-regression. Front Cardiovasc Med. (2021) 8:787270. 10.3389/fcvm.2021.787270 34977192 PMC8714841

[30] Dukkipati SR Neuzil P Skoda J Petru J d’Avila A Doshi SK Visual balloon-guided point-by-point ablation: reliable, reproducible, and persistent pulmonary vein isolation. Circ Arrhythm Electrophysiol. (2010) 3:266–73. 10.1161/circep.109.933283 20504945

[31] De Greef Y Stroker E Schwagten B Kupics K De Cocker J Chierchia GB Complications of pulmonary vein isolation in atrial fibrillation: predictors and comparison between four different ablation techniques: results from the MIddelheim PVI-registry. Europace. (2018) 20:1279–86. 10.1093/europace/eux233 29016870

[32] Chun KRJ Perrotta L Bordignon S Khalil J Dugo D Konstantinou A Complications in catheter ablation of atrial fibrillation in 3,000 consecutive procedures: balloon versus radiofrequency current ablation. JACC Clin Electrophysiol. (2017) 3:154–61. 10.1016/j.jacep.2016.07.002 29759388

[33] Tohoku S Chen S Last J Bordignon S Bologna F Trolese L Phrenic nerve injury in atrial fibrillation ablation using balloon catheters: incidence, characteristics, and clinical recovery course. J Cardiovasc Electrophysiol. (2020) 31:1932–41. 10.1111/jce.14567 32419183

[34] Yamasaki H Aonuma K Shinoda Y Komatsu Y Masuda K Hashimoto N Initial result of antrum pulmonary vein isolation using the radiofrequency hot-balloon catheter with single-shot technique. JACC Clin Electrophysiol. (2019) 5:354–63. 10.1016/j.jacep.2019.01.017 30898239

[35] Metzner A Wissner E Lin T Ouyang F Kuck KH. Balloon devices for atrial fibrillation therapy. Arrhythm Electrophysiol Rev. (2015) 4:58–61. 10.15420/aer.2015.4.1.58 26835102 PMC4711574

[36] Heeger CH Tiemeyer CM Phan HL Meyer-Saraei R Fink T Sciacca V Rapid pulmonary vein isolation utilizing the third-generation laserballoon—the PhoeniX registry. Int J Cardiol Heart Vasc. (2020) 29:100576. 10.1016/j.ijcha.2020.100576 32642555 PMC7334810

