# Supplementary figures and images for: Bayesian network meta-analysis comparing hot balloon, laser balloon and cryoballoon ablation as initial therapies for atrial fibrillation
Source: Front Cardiovasc Med. 2023 Jul 25;10:1184467. doi: 10.3389/fcvm.2023.1184467 (PMC10407100; doi:10.3389/fcvm.2023.1184467)

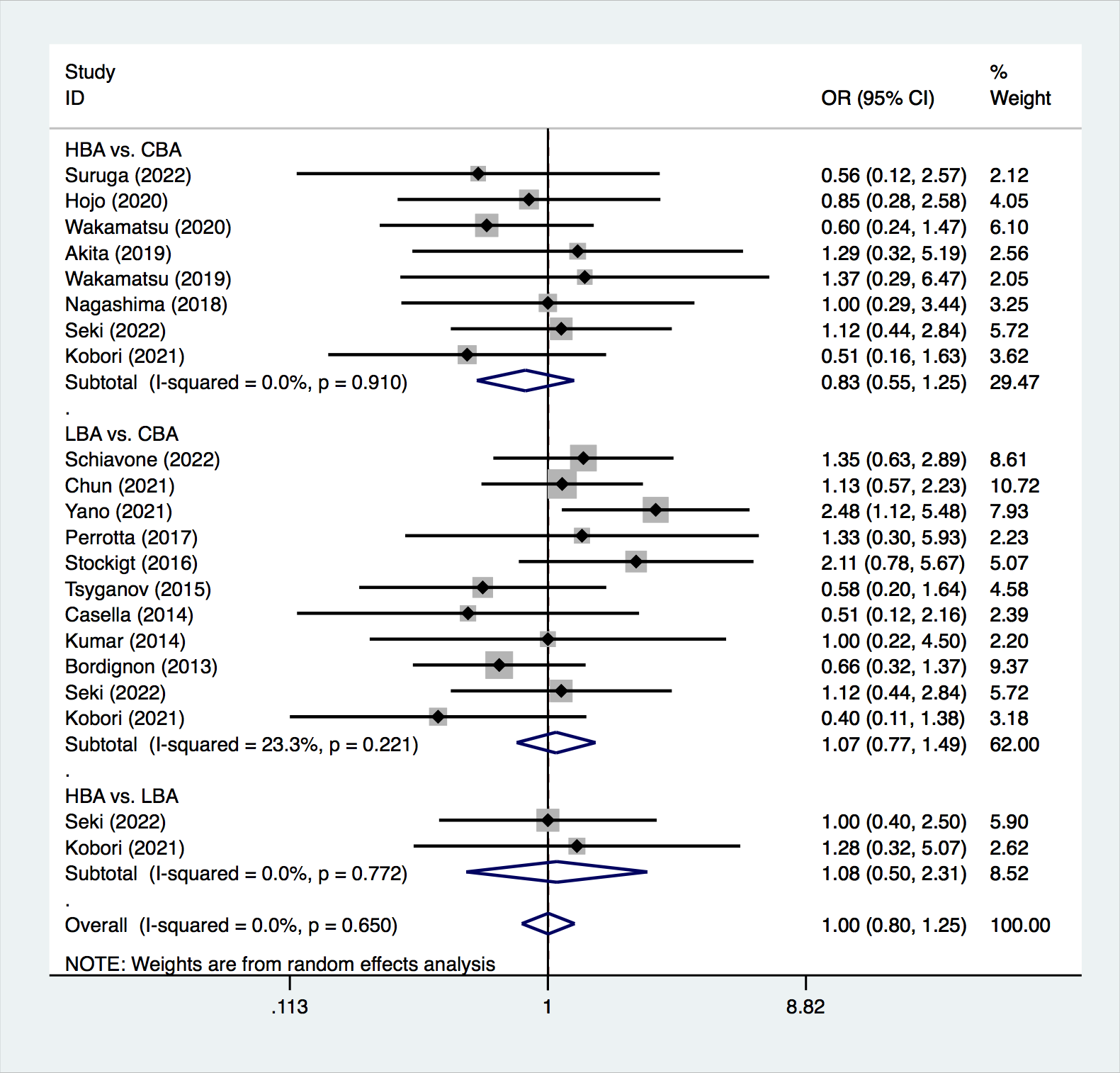

Supplement: Supplementary Figure 1 — Forest plot for the outcome of AF recurrence from pairwise meta-analysis. AF, atrial fibrillation; HBA, hot balloon ablation; LBA, laser balloon ablation; CBA, cryoballoon ablation; OR odds ratio. [file Image1.tif]

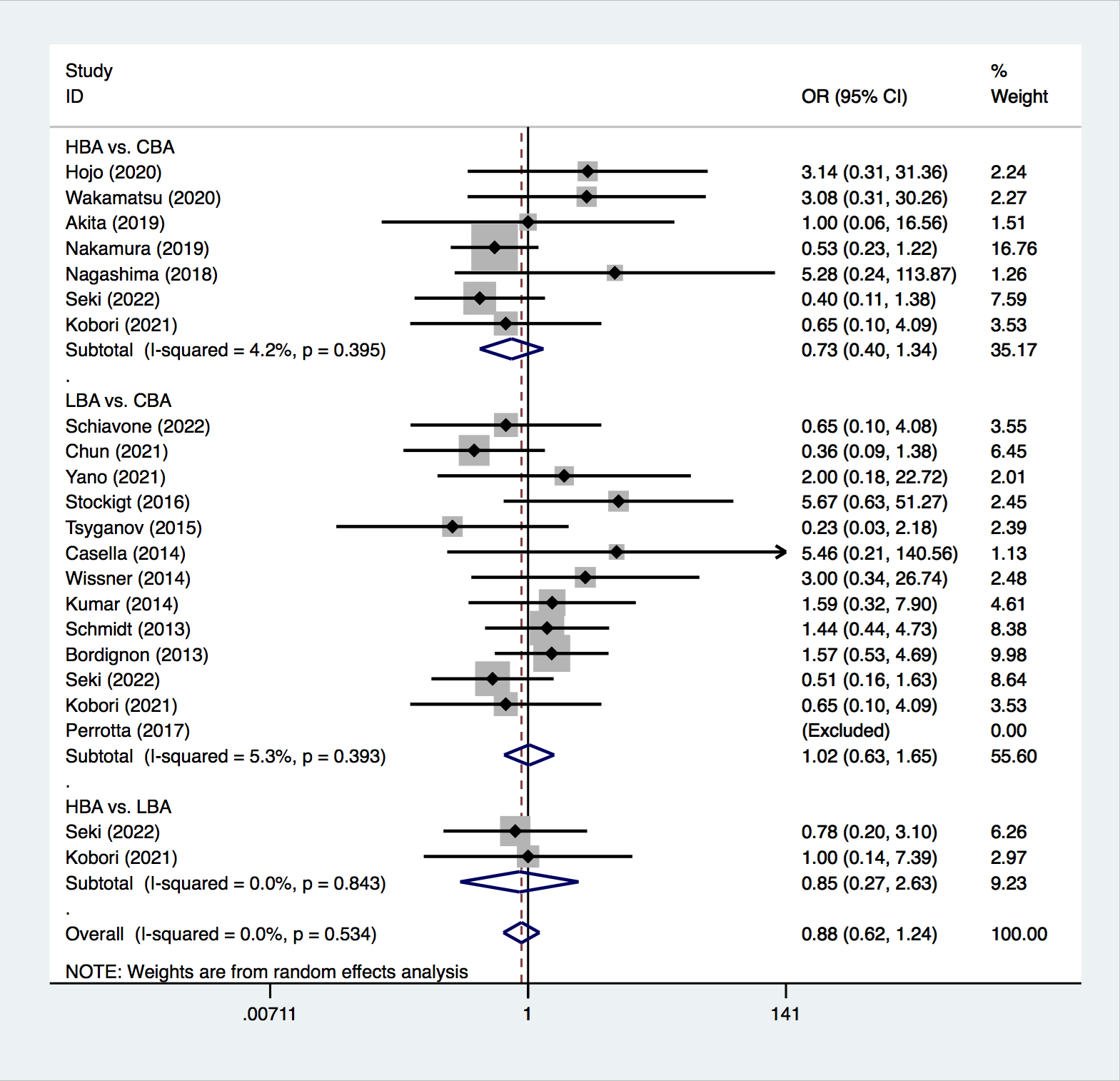

Supplement: Supplementary Figure 2 — Forest plot for the outcome of procedure-related complication from pairwise meta-analysis. HBA, hot balloon ablation; LBA, laser balloon ablation; CBA, cryoballoon ablation; OR, odds ratio. [file Image2.tif]

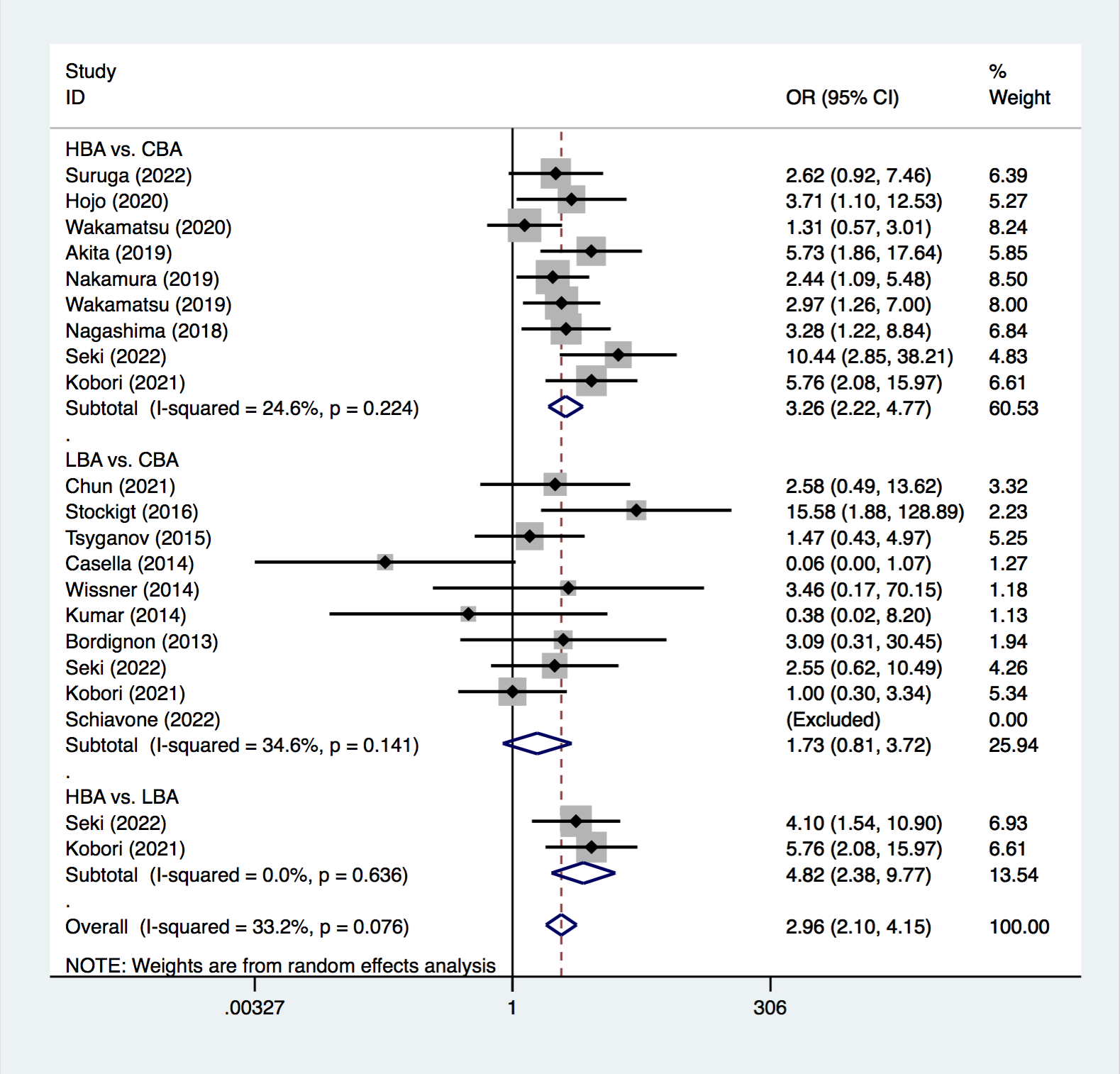

Supplement: Supplementary Figure 3 — Forest plot for the outcome of TUA from pairwise meta-analysis. HBA, hot balloon ablation; LBA, laser balloon ablation; CBA, cryoballoon ablation; OR, odds ratio; TUA, touch-up ablation. [file Image3.tif]

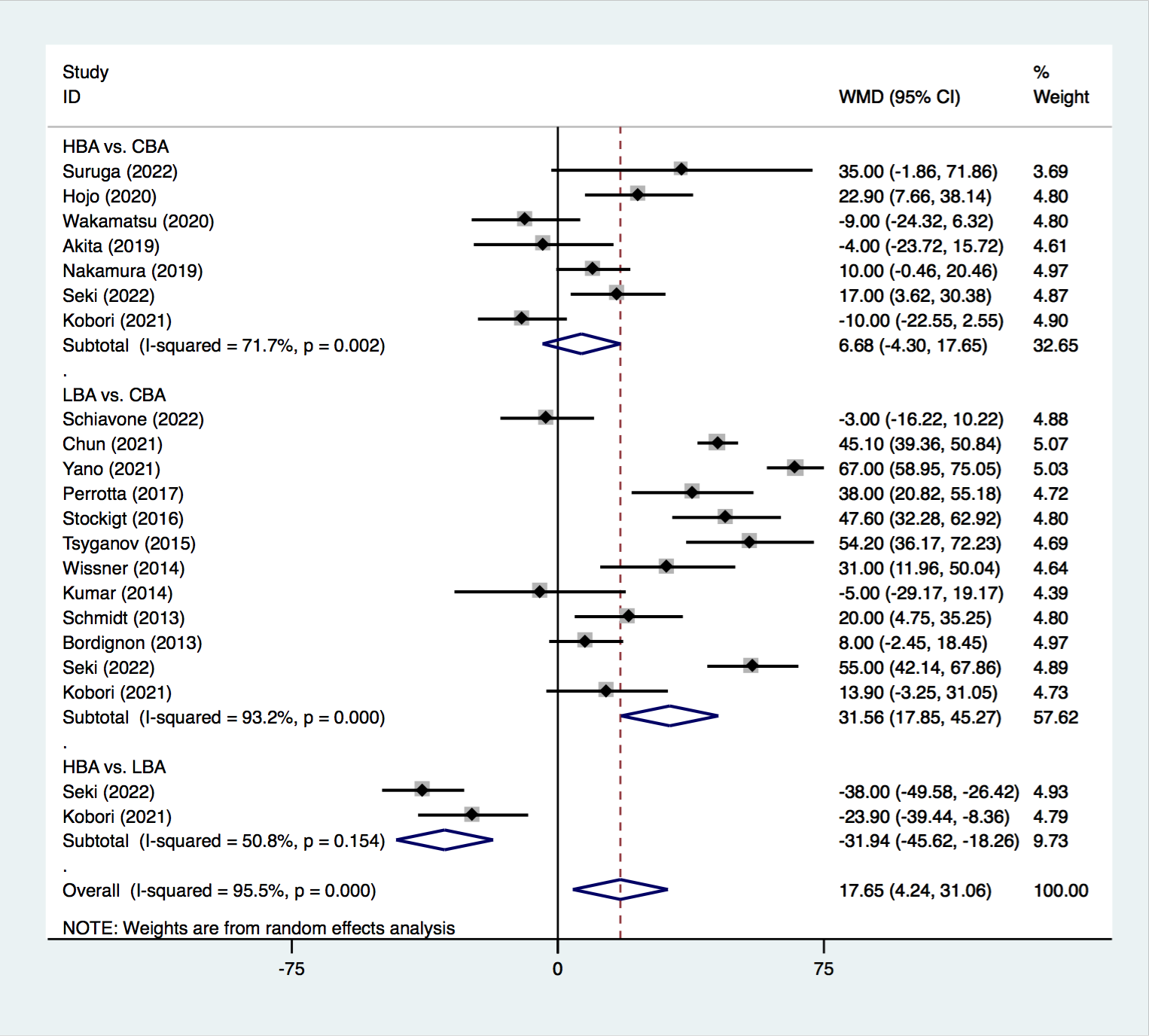

Supplement: Supplementary Figure 4 — Forest plot for the outcome of procedure time from pairwise meta-analysis. HBA, hot balloon ablation; LBA, laser balloon ablation; CBA, cryoballoon ablation; WMD, weighted mean difference. [file Image4.tif]

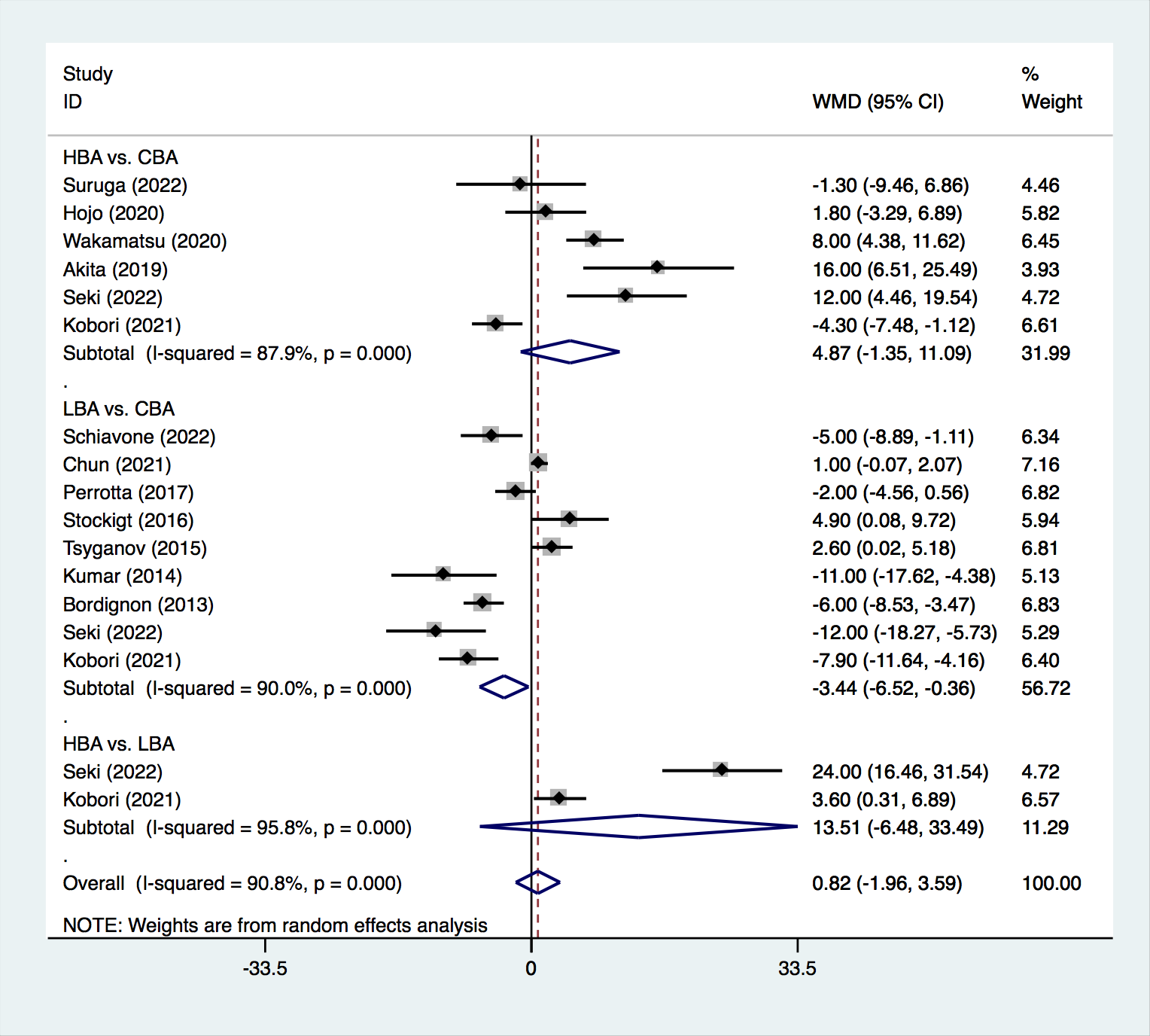

Supplement: Supplementary Figure 5 — Forest plot for the outcome of fluoroscopy time from pairwise meta-analysis. HBA, hot balloon ablation; LBA, laser balloon ablation; CBA, cryoballoon ablation; WMD, weighted mean difference. [file Image5.tif]
